# Differences in ​the proportion of children meeting behavior guidelines ​between summer ​and school by socioeconomic status and race

**DOI:** 10.1002/osp4.532

**Published:** 2021-05-26

**Authors:** Ethan T. Hunt, Lauren von Klinggraeff, Alexis Jones, Sarah Burkart, Rodrick Dugger, Bridget Armstrong, Michael W. Beets, Gabrielle Turner‐McGrievy, Marco Geraci, R. Glenn Weaver

**Affiliations:** ^1^ Department of Exercise Science University of South Carolina Columbia South Carolina USA; ^2^ Department of Health Education and Behavior University of South Carolina Columbia South Carolina USA; ^3^ Sapienza – University of Rome MEMOTEF Department Rome Italy; ^4^ Department of Epidemiology and Biostatistics University of South Carolina Columbia South Carolina USA

**Keywords:** children, physical activity, sleep, summer

## Abstract

**Objective:**

Children who fail to meet activity, sleep, and screen‐time guidelines are at increased risk for obesity. Further, children who are Black are more likely to have obesity when compared to children who are White, and children from low‐income households are at increased risk for obesity when compared to children from higher‐income households. The objective of this study was to evaluate the proportion of days meeting obesogenic behavior guidelines during the school year compared to summer vacation by race and free/reduced priced lunch (FRPL) eligibility.

**Methods:**

Mixed‐effects linear and logistic regressions estimated the proportion of days participants met activity, sleep, and screen‐time guidelines during summer and school by race and FRPL eligibility within an observational cohort sample.

**Results:**

Children (n = 268, grades = K − 4, 44.1%FRPL, 59.0% Black) attending three schools participated. Children's activity, sleep, and screen‐time were collected during an average of 23 school days and 16 days during summer vacation. During school, both children who were White and eligible for FRPL met activity, sleep, and screen‐time guidelines on a greater proportion of days when compared to their Black and non‐eligible counterparts. Significant differences in changes from school to summer in the proportion of days children met activity (−6.2%, 95CI = −10.1%, −2.3%; OR = 0.7, 95CI = 0.6, 0.9) and sleep (7.6%, 95CI = 2.9%, 12.4%; OR = 2.1, 95CI = 1.4, 3.0) guidelines between children who were Black and White were observed. Differences in changes in activity (−8.5%, 95CI = −4.9%, −12.1%; OR = 1.5, 95CI = 1.3, 1.8) were observed between children eligible versus uneligible for FRPL.

**Conclusions:**

Summer vacation may be an important time for targeting activity and screen‐time of children who are Black and/or eligible for FRPL.

## 1 INTRODUCTION

There is growing evidence that the socioeconomic status of a child's family is a key risk factor for becoming obese.^1^, ^2^ Compelling evidence exists that school‐aged children^3^, ^4^, ^5^, ^6^, ^7^ and adults^8^, ^9^ from low‐income families are at elevated risk for obesity. Recent data from the National Health and Nutrition Examination Survey show that 20% of children from families with a household income at ≤130% of the federal poverty level have obesity, while only 10% of children from families with incomes ≥350% of the federal poverty level have obesity.^10^ Importantly, this gap has increased over time. Independent of income, children who are Black are at an increased risk for obesity when compared to children who are White.^5^, ^11^ This is most likely due to the well‐documented effects of structural racism on health behaviors which underlie health disparities.^12^

Summer vacation from school is a critically important time for addressing obesity. A large body of evidence indicates that body mass index (BMI) gain accelerates during the summer.^13^, ^14^, ^15^, ^16^, ^17^, ^18^ Further, at least one study has shown that the prevalence of children with obesity increases during the months of summer.^15^ This acceleration in BMI may be due to engagement in unhealthy behaviors during the summer. For instance, a growing number of studies demonstrate that children engage in less physical activity, spend more time sedentary, and spend more time on screens during the summer than during the school year.^19^, ^20^, ^21^ Studies are also emerging that show children engage in healthier amounts of sleep and less variable sleep on nights prior to school days, compared to extended breaks from school, like summer.^20^, ^21^, ^22^ The degradation of health behaviors during summer vacation likely leads to decreased rates of meeting activity,^23^ sleep,^24^ and screen‐time guidelines.^25^, ^26^ Failing to meet these guidelines has been associated with increased risk for obesity, insulin resistance, cardiovascular and other diseases.^27^

The structured days hypothesis,^28^ which posits that structure, defined as a pre‐planned, segmented, and adult‐supervised compulsory environment, plays a protective role for children against unhealthy behaviors and, ultimately, prevents the occurrence of negative health‐outcomes, such as excessive BMI gain. The structured days hypothesis draws upon concepts in the ‘filled‐time perspective’ literature, which posits that time filled with favorable activities cannot be filled with unfavorable activities.^29^ This perspective leads to the hypothesis that children engage in a greater number of unhealthy behaviors that lead to increased BMI gain during times that are less‐structured (e.g., summer days) than during times that are more structured (e.g., school days). Correspondingly, the Health Gap Hypothesis posits that children from low‐income households and children who are Black have relatively less access to structured summer programming (e.g., summer camps) than their middle‐to‐high income and White counterparts due to financial barriers and insufficient community resources.^30^ Thus, summer may disproportionately impact the health behaviors of children from low‐income and Black households and ultimately lead to greater accelerated summer BMI gain in these children. Indeed preliminary evidence suggests that children who are Black and children from low‐income households experience greater increases in summer BMI gain compared to other children.^31^ Ultimately, greater accelerated summer BMI gain may partially explain the disproportionate risk for obesity born by children from low‐income and Black households.

The purpose of this study was to examine the proportion of days children met guidelines for moderate‐to‐vigorous physical activity (MVPA≥60 min/day),^23^ sleep (10–13 h/night for 5 year olds, 9–12 h/night for 6–12 year olds),^24^ and screen‐time (<2 h/day)^25^, ^26^ during the school year compared to the summer, and to examine if these rates differed by race and free/reduced priced lunch (FRPL) eligibility, a proxy of household income. It is hypothesized that (1) during the summer all children will meet physical activity, sleep, and screen‐time guidelines on fewer days than during the school year, and (2) children who are eligible for FRPL and children who are Black will experience greater declines in the number of days that they meet physical activity, sleep, and screen‐time guidelines than children who are not eligible for FRPL and children who are White.

## 2 METHODS

### 2.1 Study sample and design

This study utilized data from a larger natural experiment that examined changes in BMI and fitness during the summer vacation and school year for children attending a year‐round school and two match paired traditional schools.^32^, ^33^ Physical activity, sleep, and screen‐time behavioral data were collected on a subset of (n = 267) children participating in the larger study from Spring 2018–Fall 2019. This study presents obesogenic behavior data from school years (2017–2018, 2018–2019) and summers (2018, 2019). All kindergarten through third grade students participating were invited to participate in the behavioral data collection in the Spring of 2018. Measurements commenced in the spring semester of 2018 (i.e., April) and were completed in the fall academic semester of 2019 (i.e., August). Data collection occurred during three distinct one‐month measurement periods while school was in session (March and October 2018, and March 2019) and two distinct three‐month periods during the traditional summer vacation (May to August 2018 and 2019). For children in the traditional school, summer vacation lasted 11 weeks while summer vacation lasted 5 weeks for children in the year‐round school. Prior to the completion of any measures a consent letter was sent home to parents describing study procedures. Parents who consented were asked to sign and return the letter. All protocols were approved by the University of South Carolina Institutional Review Board prior to enrollment of the first participant.

### 2.2 Measures

#### 2.2.1 Race

Parents reported child's race on a single item screener once at enrollment into the study. Children whose parents reported a race other than White or Black were excluded from the current analysis (n = 26).

#### 2.2.2 Physical activity and sleep

Details fully describing the study can be found elsewhere.^33^ Physical activity and sleep were measured using a Fitbit Charge 2^TM^ (Fitbit Inc.). Fitbits were chosen because they provide good agreement with polysomnography and electrocardiography,^34^ they use multiple heartrate and actigraphy channels to classify sleep which is superior to a single‐channel actigraphy,^35^ can be charged at home, and data is stored in the cloud allowing for data collection over extended periods of time (e.g., 3‐months summer vacation). Data processing for physical activity and sleep were informed by the International Study of Childhood Obesity, Lifestyle and the Environment data processing protocols.^36^ For this analyses, only nocturnal sleep was considered. A valid night of sleep was considered sleep onset that occurred between 5 PM and 6 AM and lasted for greater than 240 min.^37^ If sleep segments were separated by less than 20 min they were considered one continuous sleep segment.^36^ Sleep duration was identified as the number of minutes that the Fitbit device classified a child as asleep during a sleep episode. To distill heartrate into activity intensity levels, each child's resting heartrate was calculated as the lowest mean beats‐per‐minute for 10 consecutive minutes each day.^38^ Heartrates were distilled into activity intensity levels based on percent heart rate reserve (HRR). Intensity levels are classified as follows: 0.0–19.9% HRR equaled sedentary, 20.0–49.9% of HRR equaled light physical activity, and ≥50.0% equaled MVPA.^39^ An individual day of at least 10 h of waking wear was considered a valid day.^36^

#### 2.2.3 Screen‐time

Screen‐time was assessed via parent proxy‐report. Parents completed a questionnaire with their child/children to report their children's screen‐time twice per week during measurement periods. Parents were asked to report on their child's daily screen‐time on at least 4 days during each 30‐days collection period. Parents/children estimated total amount of time (hours and minutes) children spent in front of a screen that day (e.g., TV, computer, video game, smartphone, and tablet).

#### 2.2.4 Household income

Poverty‐to‐income ratio (PIR) was used as a measure of household income. PIR is the ratio of household income to poverty and is calculated by dividing the total reported household income by the Department of Health and Human Services' poverty level.^40^ Parents/guardians were asked to select a household income as a single item in $10,000 increments. For this analysis, PIR was dichotomized by FRPL status according to the National School Lunch Program.^41^ Children living in a household with a PIR < 1.85 were classified as eligible to receive FRPL and a PIR ≥ 1.85 was classified as not eligible to receive FRPL.

#### 2.2.5 Statistical analyses

First, means and standard deviations of school and child characteristics were examined. Subsequently, regression analysis was used to assess the difference between meeting guidelines (dependent variable) on a school or summer day (independent variable). For each behavior, the dependent variable was operationalized as a binary variable (meeting vs. not meeting the guidelines) or as the proportion of days a child met guidelines. The independent variable was also binary (i.e., school or summer day). Multi‐level mixed effect logistic and linear regressions, respectively, were conducted to account for clustering (i.e., days nested within children and children nested within schools). One set of models included race and race‐by‐condition interactions while a second set of models included FRPL status (<1.85 PIR vs. ≥1.85 PIR) and FRPL‐by‐condition interactions. All models were adjusted for sex and grade. Analyses exploring the proportion of days children met guidelines by FRPL status included race as a covariate, and models estimating the proportion of days children met guidelines by race included FRPL status as a covariate. Analyses were carried out in Stata (v14.2, College Station TX).

## 3 RESULTS

Characteristics of the participating children are presented in Table 1 **.** A total of 267 children participated in the study with 58.1% identifying as Black and 33.0% identifying as White. A total of 51.3% of the participants identitified as female and 44.4% eligible for FRPL. During the school year children engaged in 77.6 (SD = 73.7), 470.2 (SD = 68.2), and 100.2 (SD = 89.0) minutes of MVPA, sleep, and screen‐time, respectively. During the summer children engaged in 75.3 (SD = 90.7), 486.3 (SD = 91.7), and 145.3 (SD = 120.2) minutes of MVPA, sleep, and screen‐time, respectively.

**TABLE 1:** Participant demographic and behavioral data

	(N)	%
	**267**	**100**
**Race**		
Black	155	58.1
White	86	33.0
Other	26	7.9
**Sex**		
Boys	137	48.7
Girls	130	51.3
**Grades**		
Kindergarten	17	6.4
1st	56	21.0
2nd	78	29.2
3rd	75	28.1
4th	42	15.7
**Income level**		
Eligible for FRPL (≤1.85 PIR)	120	44.1
Not eligible for FRPL (>1.85 PIR)	147	55.9
**Total weekday behavior data**	**Minutes**	**±**
Moderate‐to‐vigorous physical activity (n = 267) (n = 14,172 child days)	79.4	77.0
Sleep (n = 209) (n = 4927 child days)	474.7	75.3
Screen‐time (n = 195) (n = 2831 child days)	120.8	105.9

Abbreviation: FRPL, free/reduced price lunch.

Model implied within‐ and between‐group estimates (including covariates) of the proportion of days and odds of meeting MVPA, sleep, and screen‐time guidelines during summer vacation and the school year are presented in Figure 1a, 1b. Children who are Black were less likely to meet MVPA guidelines in the summer compared to the school year (change = −6.2% [95CI = −8.7%, −3.7%]; OR = 0.7 [95CI = 0.6, 0.8]) while children who are White were just as likely to meet MVPA guidelines during the summer compared to the school year (change = 0.0% [95CI = −3.0%, −3.0%]; OR = 1.0 [95CI = 0.9, 1.2]). This translated to children who are Black experiencing a −6.2% ([95CI = −10.1%, −2.3%]; OR = 0.7 [95CI = 0.6, 0.9]) greater decline in MVPA guideline adherence from school to summer when compared to children who are White. Children who are Black were more likely to meet sleep guidelines in the summer compared to the school year (change = 17.0% [95CI = 13.8%, 20.1%]; OR = 3.9 [95CI = 3.0, 5.0]) while children who are White were also more likely to meet sleep guidelines during the summer compared to the school year (change = 9.5% [95CI = 5.9%, 13.2%]; OR = 1.9 [95CI = 1.4, 2.5]). This translated to children who are Black experiencing a 7.4% ([95CI = 2.6%, 12.2%]; OR = 2.0 [95CI = 1.4, 2.9]) greater increase in sleep guideline adherence from school to summer when compared to children who are White. Children who are Black were less likely to meet screen‐time guidelines in the summer compared to the school year (change = −21.4% [95CI = −24.8%, −17.0%]; OR = 0.3 [95CI = 0.2, 0.4]) while children who are White were also less likely to meet screen‐time guidelines during the summer compared to the school year (change = −19.4% [95CI = −24.8%, −14.0%]; OR = 0.3 [95CI = 0.2, 0.5]). This translated to no statistically significant difference in school to summer change between children who are Black and children who are White in screen‐time guideline adherence (difference in change = −2.0% [95CI = −9.0%, 5.0%]; OR = 0.8 [95CI = 0.5, 1.2]).

**FIGURE 1a:**
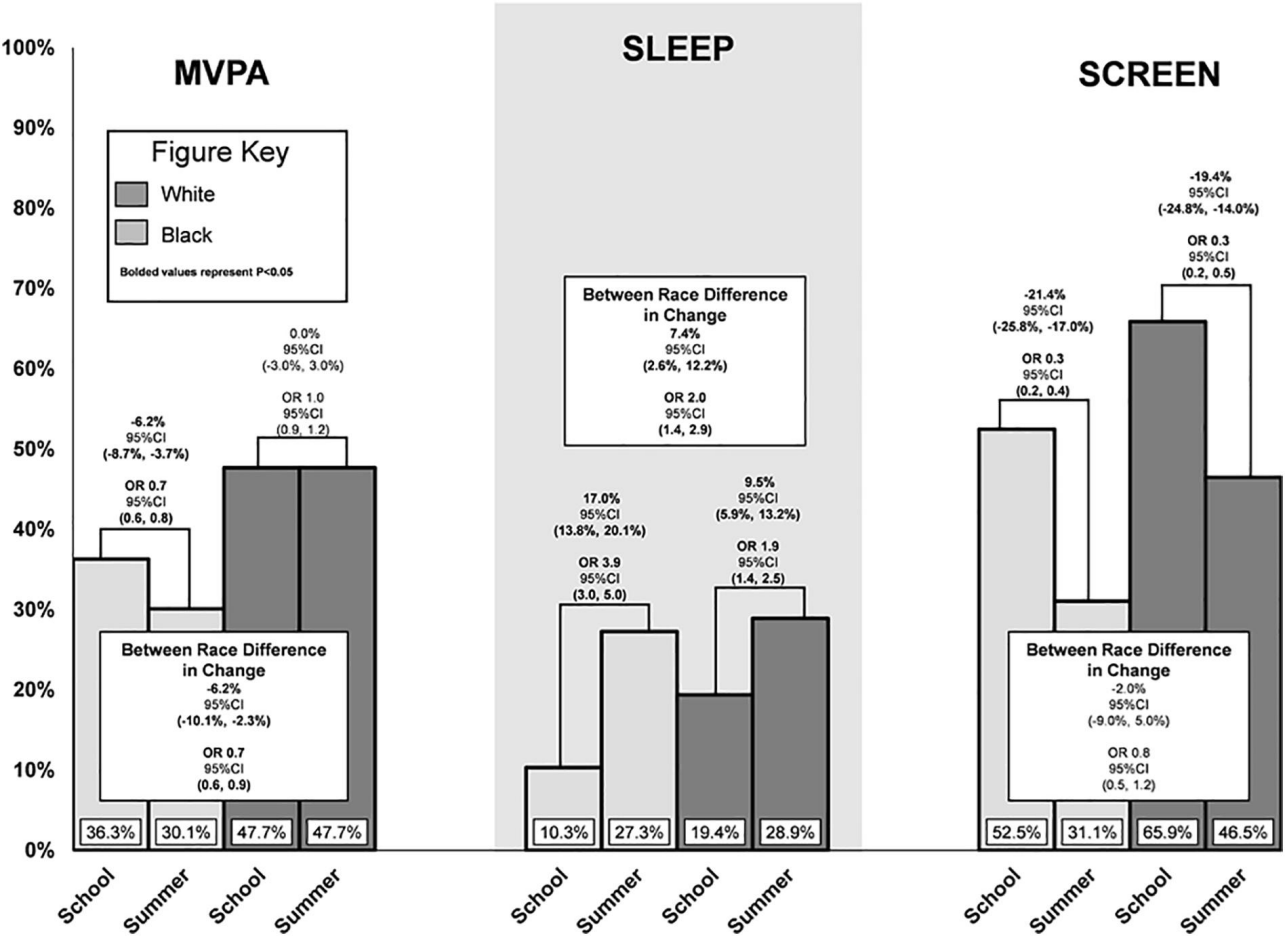
Proportion of days meeting guidelines on school days and summer vacation by race

**FIGURE 1b:**
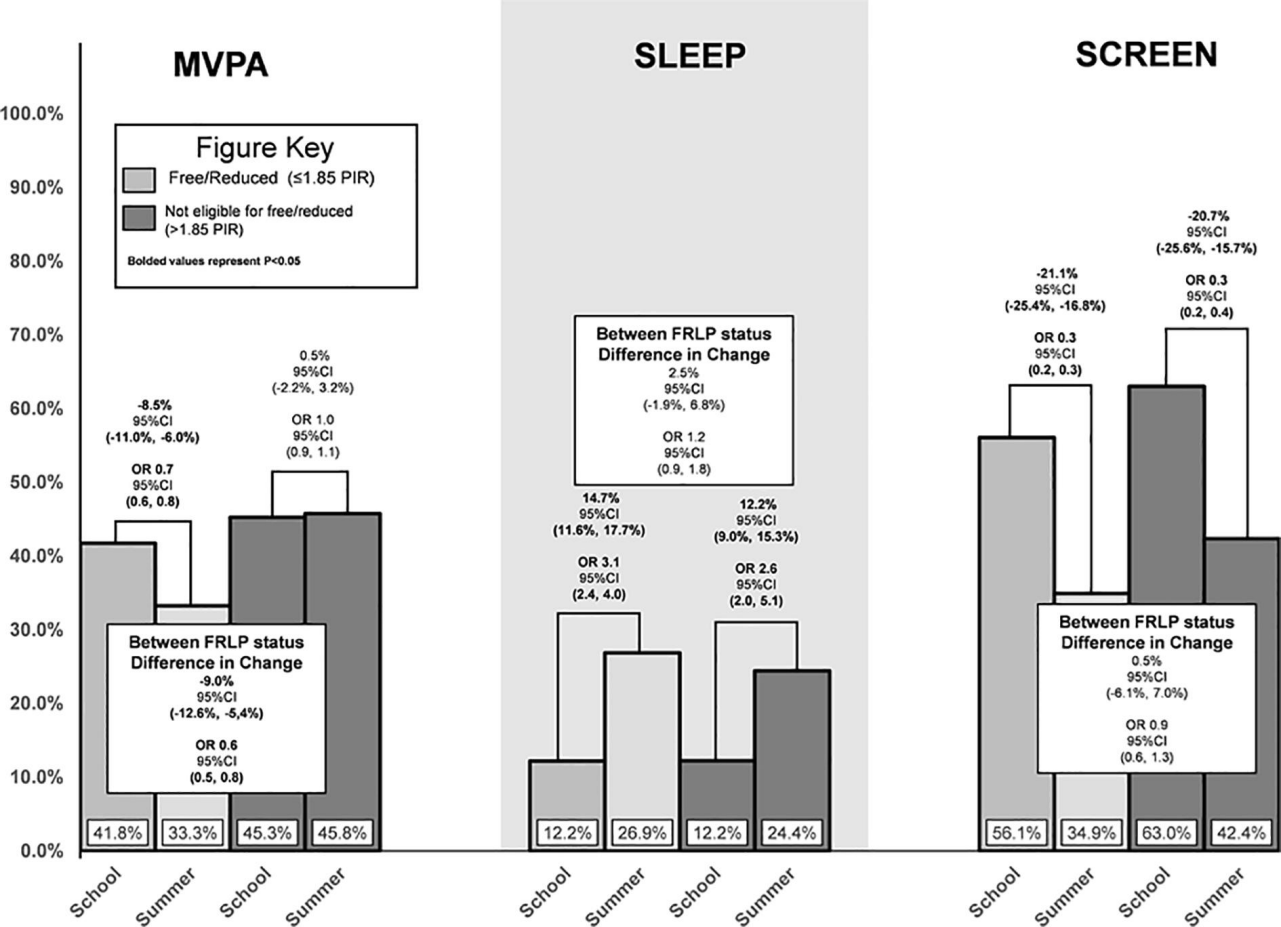
Percent of days meeting guidelines on school days and summer vacation by free/reduced status

Children eligible for FRPL were less likely to meet MVPA guidelines in the summer compared to the school year (change = −8.5% [95CI = −11.0%, −6.0%]; OR = 0.7 [95CI = 0.6, 0.8]) while children not eligible for FRPL were just as likely to meet MVPA guidelines during the summer compared to the school year (change = 0.5% [95CI = −2.2%, −3.2%]; OR = 1.0 [95CI = 0.9, 1.1]). This translated to children who are eligible for FRPL experiencing a −9.0% ([95CI = −12.6%, −5.4%]; OR = 0.6 [95CI = 0.5, 0.8]) greater decline in MVPA guideline adherence from school to summer when compared to children not eligible for FRPL. Children eligible for FRPL were more likely to meet sleep guidelines in the summer compared to the school year (change = 14.7% [95CI = 11.6%, 17,7%]; OR = 3.1 [95CI = 2.4, 4.0]) while children not eligible for FRPL were also more likely to meet sleep guidelines during the summer compared to the school year (change = 12.2% [95CI = 9.0%, 15.3%]; OR = 2.6 [95CI = 2.0, 5.1]). This translated to children eligible for FRPL experiencing no statistically significant greater increase in sleep guideline adherence from the school to summer compared to children not eligible for FRPL (difference in change = 2.5% [95CI = −1.9%, 6.8%]; OR = 1.2 [95CI = 0.9, 1.8]). Children eligible for FRPL were less likely to meet screen‐time guidelines in the summer compared to the school year (change = −21.1% [95CI = −25.4%, −16.8%]; OR = 0.3 [95CI = 0.2, 0.3]) while children not eligible for FRPL were also less likely to meet screen‐time guidelines during the summer compared to the school year (change = −20.7% [95CI = −25.6%, −15.7%]; OR = 0.3 [95CI = 0.2, 0.4]). This translated to no statistically significant difference in school to summer change between children eligible for FRPL and children not eligible for FRPL (difference in change = 0.5% [95CI = −6.1%, 7.0%]; OR = 0.9 [95CI = 0.6, 1.3]).

## 4 DISCUSSION

In the current study, all children met MVPA and screen‐time guidelines on fewer days and sleep guidelines on more days during the summer when compared to the school year. However, children who are Black and eligible for FRPL saw larger decreases in the proportion of days they met MVPA guidelines during the summer than children who are White and not eligible for FRPL. Further, children who are Black saw a larger increase in the proportion of days they met sleep guidlelines during the summer when compared to their White counterparts.

The findings of the current study align with past studies that have found children are less active and engage in more screen‐time during periods of less structure (i.e., summer, weekends, or holidays).^20^, ^28^, ^33^, ^42^, ^43^, ^44^, ^45^ Given that children who are Black and children from low‐income households experience more dramatic accelerations in BMI during the summer than their White and middle‐to‐high‐income counterparts,^31^ the finding in this study that the summer negatively impacted the MVPA of children who are Black and eligible for FRPL to a greater degree than children who are White or not eligible for FRPL is important. This finding suggests a specific behavioral mechanism, decreased MVPA, that may partially explain the greater increases in BMI gain during the summer for children who are Black and eligible for FRPL. Future interventions that target increasing the MVPA of children who are Black and children from low‐income households during the months of summer may be warranted.

While the percent of children meeting sleep guidelines was low, it is consistent with past studies that have examined sleep guideline adherence with objective measures.^46^, ^47^ Further, it is not surprising that children met sleep guidelines on more days during the summer when compared to the school year. Past studies have shown that children's total sleep time increases on weekends compared to school days and during the summer when compared to the school year.^21^, ^33^, ^48^, ^49^ However, these same studies show that children's bedtimes and wake times shift later and become more variable during the summer. While meeting sleep duration guidelines is protective against developing obesity,^50^, ^51^ sleep timing (i.e., late to bed, late to wake) and stability (i.e., keeping bed and wake time constant) have also been shown to be independent risk factors for obesity.^52^ If children's sleep timing is shifted and becoming more variable during the summer in the current sample, the benefits of meeting sleep duration guidelines may be nullified by later and more variable sleep timing.

Given the findings of the current study coupled with evidence from past studies that show children engage in behaviors that negatively impact their weight status during the summer, intervention strategies to improve children's behaviors during summer are warranted, especially for children who are Black and/or eligible for FRPL. One possible public health strategy is to provide increased access to healthy structured summer programming. At least one study has tested the impact of providing children with access to structured summer programming. Children (n = 94) were randomly assigned to either attend a structured summer camp or to experience a typical summer with no access to a structured program.^53^ Children assigned to attend the summer program lost 0.03 BMI z‐score units while those assigned to not attend gained 0.07 BMI z‐score units over the summer. While the differences were not statistically significant they trended in the expected direction. Further children assigned to attend the summer program engaged in 2.3% more MVPA during the program compared to the school year while children not attending the program engaged in 1.9% less MVPA during the summer compared to the school year. This pilot study shows promise for the strategy of providing structured summer programming to enhance health behaviors and mitigate accelerated summer BMI gain.

This study has several strengths including the collection of data continuously for 30+ days during the school year and summer vacation, the within‐person design (i.e., same children measured during the school year and summer), and the grounding in theoretical frameworks (i.e., Structured Days Hypothesis and Health Gap Hypothesis). This study also has limitations that must be considered when interpreting the results. First, this study only included three schools in the southeastern United States. Thus, the generalizability of findings may be limited. Second, one of the three schools followed a year‐round calendar. Thus, there may be systematic differences in the findings between school calendar types. Third, the study used Fitbit devices to quantify physical activity and sleep. While these devices have shown good agreement with electrocardiography assessment of heartrate and polysomnography assessment of sleep,^34^, ^54^, ^55^ they have been sparsely used in physical activity and sleep. This limits the ability to compare the findings of this study with other studies.

## 5 CONCLUSIONS

During summer, children are less likely to meet guidelines for physical activity and screen‐time, providing partial support for the Structured Days Hypothesis.^28^ This is particularly true for children who are Black or eligible for FRPL, providing support forthe Health Gap Hypothesis.^30^ Interventions that target MVPA and screen‐time during times of less structure (i.e., summer), may be warranted.

## CONFLICT OF INTEREST

The author declares no conflict of interest.

## AUTHORS CONTRIBUTIONS

Ethan T. Hunt and R. Glenn Weaver carried out the project. Ethan T. Hunt wrote the manscript with support from Lauren von Klinggraeff, Alexis Jones, Sarah Burkart, Rodrick Dugger, Bridget Armstrong, Michael W. Beets, Gabrielle Turner‐McGrievy, Marco Geraci, and R. Glenn Weaver. R. Glenn Weaver helped supervise the project.
